# Homozygous Pathogenic MYH3 Variants Associated With Arthrogryposis and Lingual Dystonia

**DOI:** 10.5334/tohm.1079

**Published:** 2025-10-27

**Authors:** Charlotte Mouraux, Claire Fouquet, Keith Durkin, Vinciane Dideberg, Saskia Bulk, David Aktan, Maria Artesi, Frédérique Depierreux

**Affiliations:** 1 GIGA – CRC Human Imaging – Rare Movement Disorders Research Group, University of Liège, Liège, Belgium; 2 Department of Human Genetics, University Hospital of Liège, Liège, Belgium; 3 Laboratory of Human Genetics, GIGA-Institute, University of Liège, Liège, Belgium

**Keywords:** genome sequencing, pediatric movement disorders, focal dystonia, tongue dystonia arthrogryposis, dysmorphology

## Abstract

**Introduction:**

Heterozygous pathogenic variants in *MYH3* are known to be responsible for distal arthrogryposis.

**Case report:**

We report a consanguineous family of four children with two likely pathogenic *MYH3* homozygous variants associated with complex movement disorders, especially prominent lingual dystonia, along with skeletal abnormalities. The two variants in *MYH3* (c.3445G>A and c.4760T>C) have already been described in patients with congenital arthrogryposis. No other significant variation was found using long-read whole genome sequencing.

**Discussion:**

We have extended the phenotype of *MYH3*-associated arthrogryposis to include movement disorders, which may have been underdiagnosed to date.

**Highlights:**

This article extends the phenotype of *MYH3*-associated arthrogryposis to include movement disorders, illustrating a family of four children presenting *MYH3* skeletal disorders and lingual dystonia. Two homozygous likely pathogenic variants have been identified in the four sibs and appear to be causative for both skeletal and neurological phenotypes.

## Introduction

Dystonia is a hyperkinetic movement disorder characterized by sustained or intermittent muscle contractions that cause abnormal, often repetitive, movements, postures, or both [1]. Lingual dystonia is a rare form of focal dystonia involving the lingual muscles [2,3]. Its prevalence is estimated to be around 0.6 per million [4]. This subtype of oromandibular dystonia has disabling consequences as it interferes with speaking, chewing and swallowing [4,5]. The involuntary movements of the tongue vary from intermittent tongue protrusion to curling of the tongue induced by speaking [4,5]. Tongue dystonia may be idiopathic, sporadic or related to secondary causes, such as brain lesions (e.g., stroke, encephalitis or brain traumatic injury) or iatrogenic, including neuroleptic intake (i.e., tardive syndrome) [5]. Lingual dystonia has also been described in genetic conditions such as neurodegeneration with brain iron accumulation (NBIA), Wilson disease, chorea-acanthocytosis or metabolic diseases, including Lesch-Nyhan syndrome and Gaucher disease [4,5]. In these genetic disorders, the clinical picture is frequently complex, associating several types of hyperkinetic movement disorders, including dystonia.

We report a family of four children presenting severe lingual dystonia and skeletal abnormalities. To date, the association between lingual dystonia and skeletal diseases, especially arthrogryposis, has not been previously reported. All four siblings are carriers of two homozygous likely pathogenic variants in *MYH3*. Heterozygous pathogenic variants in *MYH3* are responsible for distal arthrogryposis (DA) syndromes type 2A and type 2B3 [6] which are characterized by contractures of proximal and distal joints [6]. In DA type 2A, patients also feature scoliosis and dysmorphism such as a whistling face [7]. In addition to DA, *MYH3* has been associated with contractures, pterygia and spondylocarpotarsal fusion syndrome (CPSFS) which is characterized by joint contractures and vertebral, carpal and tarsal fusions [8]. CPSFS type 1A is caused by monoallelic pathogenic variant in *MYH3* while CPSFS type 1B is caused by compound heterozygous pathogenic variants [9]. Up to now, no movement disorders have been described associated *MYH3* variants either at the monoallelic or biallelic state.

## Case description

**Figure 1:**
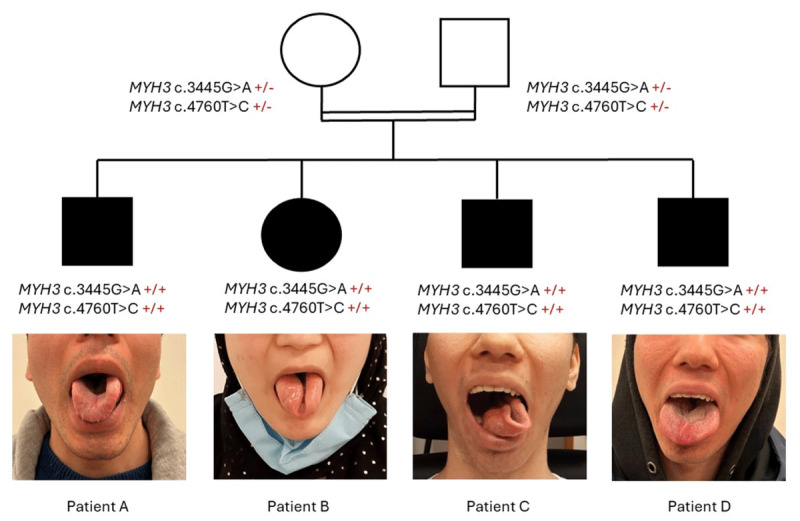
Pedigree of all four affected children presenting lingual dystonia. All children are homozygous for the two missense variants in MYH3 gene and both parents, which are asymptomatic, are heterozygous for the two variants. Each tongue picture is taken as the subject is requested to sit, at rest on the examination bench, with his mouth opened.

**Figure 2:**
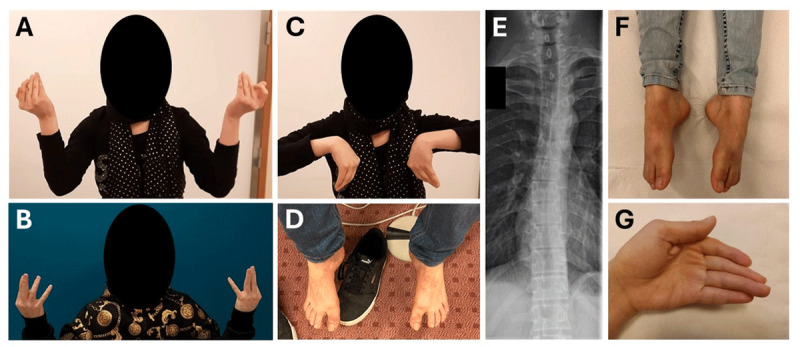
Clinical phenotype – **2A,B,C:** Hand dystonia. Pictures A and C illustrate patient B and picture B illustrates patient D. In picture A and B, patients are asked to lift their hands while sitting at rest. In picture C, patient B is asked to put her hands facing each other; **2D** – Foot dystonia in patient D while sitting at rest on the bench; **2E** – Vertebral X-ray illustrating scoliosis in patient B; **2F,G** – Feet and hands arthrogryposis in patient B.

We report a consanguineous Afghan family of four affected children with asymptomatic parents. All siblings (patients A, B, C, and D on Figure 1) present a severe lingual dystonia associated with jaw contractures resulting in dysarthria, speech delay and feeding difficulties (Figure 1). Abnormal movements of the tongue were first noticed by the mother since she tried to feed them, which suggests an onset during the first year of life. Lingual dystonia follows a torsion pattern inducing a curling of the tongue while speaking or opening the mouth (Video 1, segments 1 to 4). While patients A and C present an isolated lingual dystonia, patients B and D also present a multifocal dystonia affecting hands and feet along with a cervical dystonia in patient D (Figure 2A-D and Video 1, segments 2 and 4). Hand dystonia is characterized by a torsion pattern with prominent finger flexion, worsened by voluntary movement. Patients B, C and D have no other neurological sign. Patient A shows slight parkinsonian features, including bradykinesia, hypomimia, reduced blinking and paucity of spontaneous movements, but neither tremor nor parkinsonian gait. Gross and fine motor development were otherwise normal for all children. Routine investigations, including brain MRI and laboratory work-up, were normal for all.

**Video 1:** Neurological examination of our patients’ dystonic features. Each tongue video shows the patient first at rest and then moving voluntarily the tongue. **Segment 1:** Isolated lingual dystonia examination in patient A. Lingual dystonia follows a torsion pattern inducing a curling of the tongue while opening the mouth. **Segment 2:** Examination of patient B with lingual dystonia (2–1). Pronator drift test reveals both hand dystonia and arthrogryposis of hands. There is no cerebellar dysfunction, but finger-to-nose test is difficult to achieve because of the torsion pattern resulting from hand dystonia (2–2) When sitting on the examination bench, a varus equine position of both feet is observed, but not during gait examination. **Segment 3:** Examination of patient C, presenting with lingual dystonia. **Segment 4:** Examination of patient D with lingual dystonia (4–1). Other dystonic features are also present, with cervical dystonia with a right torticaput pattern (4–2).

All siblings present with skeletal abnormalities. Scoliosis and short stature are present in patients A, B and C (Figure 2E). Carpal, tarsal, or vertebral fusions were absent. Patient B shows obvious signs of distal arthrogryposis in hands and feet (Figure 2F-G) such as an absence of flexion creases between the intermediate and distal phalanges from the second to the fourth fingers, and a joint pterygium at the distal phalanx of the fifth finger bilaterally. Distal limb arthrogryposis is associated with dystonia in patient B, with arthrogryposis responsible for abnormal posture that is almost fixed, whereas dystonia is responsible for twisting movements with a stereotyped pattern, and is worsened by voluntary action (Video 1, segment 2). In contrast, patients A, C and D do not have distal arthrogryposis but present craniofacial dysmorphism including retrognathia. Patients C and D have low-set ears. Other features include thumb hyperlaxity in patients A, C and D and elbow hyperlaxity in all siblings.

As the family is consanguineous, we suspected a disease with a recessive transmission mode. We performed an array comparative genomic hybridization (aCGH) (Agilent, 180K chip), which did not highlight any genomic gains or losses associated with patients’ phenotype. One region of homozygosity (ROH) of approximately 6 Mb located on the chromosome 17 was detected in all four children (Chr17:10916653 to Chr17:16890221, hg19/GRCh37). We proceeded to short-read whole exome sequencing (WES) (Human Comprehensive Exome of Twist Biosciences) for all family members. We extracted all single nucleotide polymorphisms (SNPs) or insertions-deletions (indels) from WES data which were homozygous for children and heterozygous for parents. Variant prioritization, based on rarity and predicted protein effect, highlighted a shortlist of variants including two single nucleotide variants (SNVs) in *MYH3*, located within the approximate ROH previously observed by aCGH. No other candidate variant associated with patients’ phenotype was detected using WES. To exclude intronic and structural variants (SVs), we performed long-read whole genome sequencing (WGS) by nanopore (PromethION, Oxford Nanopore Technologies) for one sibling (29X coverage). No supplementary candidate variant was found in the overlapping Chr17 ROH nor in the rest of the genome. For materials and methods, see **supplementary material**.

All four children were homozygous for two missense variants (c.3445G>A,p.Glu1149Lys and c.4760T>C,p.Leu1587Pro) in *MYH3* (Figure 1). We classified the two variants following the American College of Medical Genetics and Genomics (ACMG) criteria [10] and its recent update [11] (Table 1). These two variants are rare – with an allele frequency of 0.00001067 for c.3445G>A and of 0.00001193 for c.4760T>C – with no homozygous carrier in gnomAD [12] (PM2). Computational evidence predicted a damaging effect only for c.3445G>A (i.e., REVEL score 0.777 and amino acid residue highly conserved) (PP3). Both variants segregates with the disease in this family of four affected children (i.e., homozygous affected siblings and heterozygous asymptomatic parents) (PP1_Moderate). Moreover, the evidence of skeletal abnormalities in all affected family members and the patient B’s DA, corresponding to the classical presentation of *MYH3* disorders, can be considered as a highly specific phenotype for the gene (PP4). Finally, both variants were already described together, both in the homozygous state, in two sibs with congenital arthrogryposis [13]. Consequently, both variants were classified as likely pathogenic (class IV).

**Table 1:** *MYH3* variants classification.


Variant cDNA (NM_002470.4)	**c.3445G>A**	**c.4760T>C**

Variant protein (NP_002461.2)	**p.Glu1149Lys**	**p.Leu1587Pro**

HGVS genomic (hg 19)	Chr17:g.10541644C>T	Chr17:g.10535989A>C

PM2: Rarity	GnomAD frequency = 0.00001067 with no homozygous	GnomAD frequency = 0.00001193 with no homozygous

PM3: already described	Described in Morali 2024	Described in Morali 2024

PP1: segregation	Family of 4 affected siblings	Family of 4 affected siblings

PP2: missense sensitive	Missense sensitive constraint score GnomAD = 4.67	Missense sensitive constraint score GnomAD = 4.67

PP3: in silico score	Highly conserved amino acid REVEL score = 0.777, CADD score = 5.5	Moderately conserved amino acid REVEL score = 0.482, CADD score = 5.07

PP4: phenotype	Patient B has typical distal arthrogryposis	Patient B has typical distal arthrogryposis


## Discussion

We describe a family of four affected children presenting lingual dystonia associated with skeletal abnormalities, congruous with previously reported *MYH3* skeletal disorders. All siblings carried two homozygous likely pathogenic variants in *MYH3* which seems to be causative for both skeletal abnormalities and movement disorders in this family. No other variant related to the phenotype was identified after an in-depth study of the genome using aCGH, WES and long-read WGS.

These *MYH3* missense variants have already been described together, both in the homozygous state, in two children with congenital arthrogryposis with one variant that was considered as hypomorphic (i.e., c.4760T>C) [13]. Even if the authors did not mention any movement disorder, they described some mobile appendicular posturing of the hands which is suggestive of dystonia. Besides, distinguishing dystonia from arthrogryposis requires a specific expertise in neurology, particularly in movement disorders, and those patients are frequently examined by pediatricians or rheumatologists. The prevalence of movement disorders and especially dystonia in arthrogryposis is therefore probably underestimated.

Co-segregation of the skeletal abnormalities associated with *MYH3* in affected members of this family is another convincing argument incriminating this gene. Moreover, scoliosis and short stature, presented by our patients, appear to be overlapping features in all *MYH3* patients previously reported [14,15]. The other skeletal features presented by the siblings, already described in *MYH3* disorders, include retrognathia in patients A, C and D and DA in patient B.

The pathophysiology of arthrogryposis and dystonia must be distinct, with one primary myogenic, and the other neurogenic. Besides muscles and bones, *MYH3* is also expressed postnatally in the brain, including the basal ganglia [8], a region frequently implicated in dystonia pathophysiology [16]. Functional studies previously showed that *MYH3* missense variants disturb canonical TGF-beta signaling [17]. The latter is crucial for skeletal muscle and bone development and function. Alteration of this pathway has also been reported in dystonia (e.g., in DYT-*TOR1A* [18]). Moreover, dystonia and arthrogryposis have already been associated with biallelic variants in *TOR1A* which are responsible for congenital multiplex arthrogryposis type 5 [19]. Therefore, we postulate that our *MYH3* missense variants could inhibit the TGF-beta pathway and may lead to dystonia through the inhibition of this pathway in the basal ganglia.

In conclusion, we have extended the phenotypic spectrum of *MYH3* associated disorders by reporting the first neurological features including movement disorders, especially lingual dystonia, in four new patients from the same consanguineous family. Clinicians should be aware of neurological abnormalities that might be observed in *MYH3* patients and possibly in all patients presenting DA. These patients need to be referred to specialized neurologists to consider specific treatments for their movement disorders, and especially for dystonic features, such as botulinum toxin injections or complex glossectomy [20]. This case highlights how close collaboration between neurologists and geneticists can provide critical insights into complex diagnostic challenges, particularly when exploring difficult genotype-phenotype correlations.

## Data Accessibility Statement

The data that support the findings of this study are available from the corresponding author upon reasonable request.

## Additional File

The additional file for this article can be found as follows:

10.5334/tohm.1079.s1 Supplementary material. Materials and methods used in genomic analysis.
