# Supplementary material for: Homozygous Pathogenic MYH3 Variants Associated With Arthrogryposis and Lingual Dystonia
Source: Tremor Other Hyperkinet Mov (N Y). 2025 Oct 27;15:53. doi: 10.5334/tohm.1079 (PMC12577538; doi:10.5334/tohm.1079)
Supplement: Supplementary material. — Materials and methods used in genomic analysis. [file tohm-15-1-1079-s1.pdf]

Supplementary material

Materials and Methods

Germline DNA was extracted from peripheral blood using the NucleoMag kit (Macherey-Nagel) according to the manufacturer's instructions. High-resolution microarray analysis was performed using the SurePrint G3 ISCA CGH+SNP Kit, 4x180K (Agilent) according to the manufacturer's instructions.

Short-read whole exome sequencing (WES) was carried out on DNA from peripheral blood for all family members. Enrichment was performed using the Twist Human Comprehensive Exome (Twist Bioscience) and libraries were sequenced on an Illumina NovaSeq sequencer. Alignment to the hg19/GRCh37 and variant calling (SNPS and INDELs) were done using the Burrows Wheel Aligner (BWA) (1) and the GATK toolkit respectively. Alissa Interpret (Agilent Technoligies version 5.4) was used for variant interpretation.

Long read whole genome sequencing was carried out on the Oxford Nanopore PromethION using R10.4.1 flow cells, genomic DNA was prepared via the Ligation Sequencing Kit V14 following the manufactures instruction. Basecalling was done with dorado (https://github.com/nanoporetech/dorado) using the super accurate model (dna_r10.4.1_e8.2_400bps_sup@v4.1.0). Mapping to GRCh37 was carried out with minimap2 (https://github.com/lh3/minimap2). Structural variants were called with (https://github.com/fritzsedlazeck/Sniffles) and annotated via AnnotSV (https://github.com/lgmgeo/AnnotSV). SNVs were called via Clair3 (https://github.com/HKU-BAL/Clair3) and filtered and annotated with SnpEff & SnpSift (https://pcingola.github.io/SnpEff/).

Bibliography :

1. Li H, Durbin R. Fast and accurate short read alignment with Burrows-Wheeler transform. Bioinformatics [Internet]. 2009 Jul [cited 2024 Mar 14];25(14):1754–60. Available from: https://pubmed.ncbi.nlm.nih.gov/19451168/
